# Optimizing resources in genomic surveillance: South Carolina’s QC-plus approach

**DOI:** 10.3389/fpubh.2025.1694911

**Published:** 2025-11-10

**Authors:** Rachel Cox, Gregory Goodwin, Jessica Freeman, Gabrielle Godfrey, Anyway Kapingidza, Andrew Smith, Julia Scott, Abdoulaye Diedhiou, Karla Buru, Cory J. Weaver, Ona Adair, Jenny Meredith, Chukwuemika Aroh

**Affiliations:** 1 South Carolina Department of Public Health, Columbia, SC, United States; 2 South Carolina First Steps, Columbia, SC, United States

**Keywords:** whole genome sequencing, South Carolina, variant surveillance, public health laboratory, genomic epidemiology, cycle threshold

## Abstract

Whole genome sequencing (WGS) is the gold standard for identifying emerging variants during epidemics but is resource intensive. Traditionally, a low RT-qPCR cycle threshold (Ct) is used to select samples with presumed high viral loads but whether better alternatives exist is unclear. This study introduces and evaluates a Ct-independent method, SCQC-Plus (SCQC+) approach, combining enhanced library preparation and agarose gel-based quality control for selecting samples for sequencing. From June 2022 through December 2024 at the state public health laboratory, over 1,800 SARS-CoV-2 positive clinical samples were sequenced and studied in two phases: retrospectively and prospectively. In the first phase when all PCR positive samples received into the laboratory were sequenced, we simulated the impact of two Ct-restriction thresholds (Ct < 28 and Ct < 30) by excluding those samples from the data. In the prospective phase, we tested three selection strategies on sequencing efficiency: Seq-All, Ct < 30, and SCQC+. Lastly, we compared the variants captured by a centralized state public health laboratory with those of commercial and clinical labs in the state. Results from the retrospective study suggested that Ct restriction of 30 was cost effective but missed variants in circulation. Prospectively, we found that the SCQC+ approach had a comparable cost effectiveness to Ct-restricted approach. Notably the SCQC+ approach halved the fail rate for samples with Ct over 30, resulting in the sequencing of two variants not found among samples with Ct under 30. Finally comparing the variants detected by commercial and clinical laboratories in the state identified unique variants not detected in the sampling of the state public health laboratory. This observation suggested the importance to public health of maintaining such partnerships to enable timely and comprehensive variant surveillance program. The goal of the sequencing program can impact the cost effectiveness of different approaches for sample selection. When the goal is the early detection of emerging or rare variants of concern prior to wide dispersal into the population, we propose a combination of the SCQC+ approach internally and partnership with in-state commercial and clinical laboratories, externally, as important requirements for achieving that goal.

## 1 Introduction

Real time variant surveillance by whole genome sequencing (WGS) has increasingly become an essential tool for the public health response to epidemics such as Zika, Ebola, SARS-CoV-2, and MPOX (1). SARS-CoV-2 variants with mutations impacting clinical severity, transmissibility, diagnostics, therapeutics, vaccine efficacy were identified and designated as variants of interest (VOI) or variants of concern (VOC) and prioritized for molecular surveillance (2). WGS can enable data driven decision making to limit and control morbidity and mortality in a population during an epidemic.

While WGS is the gold standard for identification of emerging variants, it is resource intensive making the sequencing of most SARS-CoV-2 positive samples impractical, even for high income countries. As such an unbiased sampling of the population of interest and sequencing of samples as efficiently as possible are required to detect VOCs/VOIs or monitor their prevalence in the community. Traditionally, a low PCR cycle threshold (Ct), typically less than 30 Ct, is used to select samples with presumed high viral loads and culturable virus (Ct-Restricted Approach) and reflex them for sequencing (3–6) but the impact this selection policy may have on the detection of rare variants remains unclear. This study introduces a Ct-independent method—SCQC+ approach—combining enhanced library preparation and agarose gel-based quality control with a goal of improving cost effectiveness and maintaining comprehensive variant detection.

Here we present a case report evaluating the cost-effectiveness of different approaches to selecting samples for sequencing and explore the contribution afforded by commercial labs to variant surveillance provided by a state public health laboratory.

## 2 Context (setting and population)

**Figure 1:**
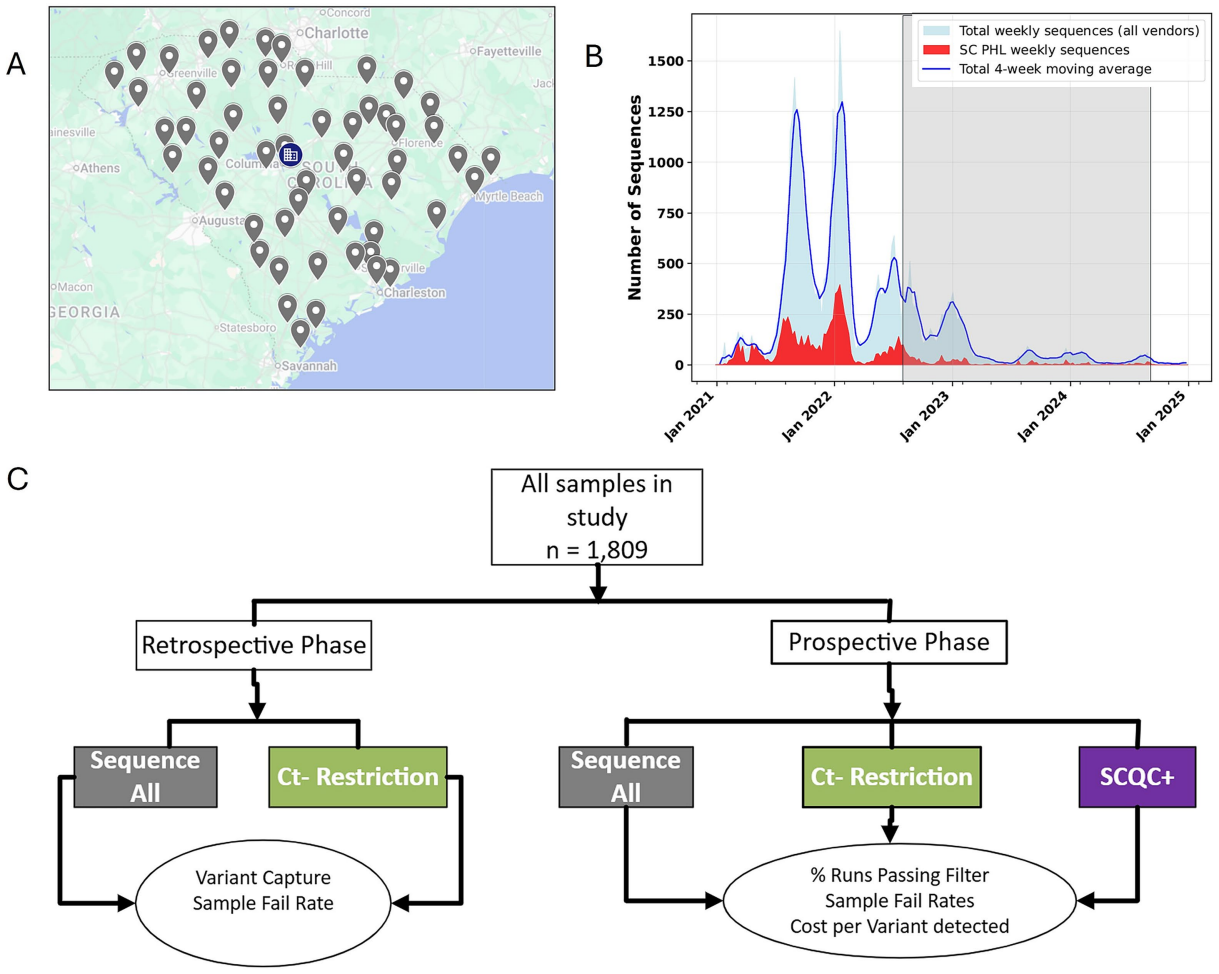
South Carolina has a centralized public health system that enables sampling of clinical cases throughout the state. **(A)** As a state with a centralized public health system, all local health department offices throughout the state, indicated by gray icons, are joined together in one surveillance network, with sequencing of samples carried out centrally at the SCPHL, as indicated by the blue building icon. **(B)** An overview of the sequencing effort of the state public health laboratory, in red, versus the total number of sequences from other sources in the state, in blue. The shaded area indicates the period of study for this publication. **(C)** An overview of the study design and its two phases aimed at determining a cost-effective approach for real time variant surveillance during a public health emergency.

State public health agencies are uniquely positioned at the front lines of epidemic response. A state public health system can either be centralized, decentralized, or hybrid. South Carolina is a state with a population of 5.4 million and represents one of 14 states or territories in the U.S. with a centralized public health system (7). As such, South Carolina’s local health departments scattered throughout the state (Figure 1A), belong to the same organization, the South Carolina Department of Public Health (DPH), and enable the statewide collection and shipping of surveillance samples to the South Carolina public health laboratory (SCPHL) (Figure 1B), a bureau within DPH. Moreover, all the sequencing of other SARS-CoV-2 positive cases by other laboratories are also reported to DPH.

To detect existing and emerging variants of SARS-CoV-2, SC PHL conducts random sampling of PCR positive samples throughout the state. To sequence samples, sample RNA are first converted to cDNA using New England Biolabs, LunaScript RT Supermix Kit. Enrichment and amplification of the cDNA library was done using Artic Primers and New England Biolabs, Q5 Hot Start High-Fidelity 2x Master Mix. For library preparation, Illumina’s DNA Prep Kit was used to perform tagmentation and post PCR clean-up for the selection of optimal DNA fragments. The DNA Prep Kit uses Bead-Linked Transposomes that bind to a limited volume of cDNA, selecting for size-specific DNA fragments. The result is a pooled sample of ideal-sized DNA strands. Libraries were sequenced on Illumina MiniSeq or MiSeq platforms, producing FASTQ sequence files. Analysis was achieved using Illumina’s BaseSpace App, DRAGEN COVID Lineage, which used the Wuhan-Hu-1 reference to analyze pathogen data and create consensus FASTAS.

For analysis of sequencing efforts throughout the state, SARS-CoV-2 variant surveillance data were obtained from DPH surveillance database covering the period from January 1, 2021, to January 1, 2025. Data originated from South Carolina’s multi-laboratory surveillance network including SCPHL, commercial diagnostic laboratories (Quest Diagnostics, LabCorp, Aegis Sciences Corporation, Mako Medical Laboratories), academic medical centers (Medical University of South Carolina), and other participating healthcare facilities. To ensure quality assurance duplicate and statistical independence, entries were identified and removed using case identification numbers. Data validation included verification of specimen collection dates, facility identifiers, and variant nomenclature consistency. The final analytical dataset comprised of 43,150 unique, successfully sequenced SARS-CoV-2 cases with metadata. Statewide analyses were implemented in Python 3.x using established scientific computing libraries: pandas (≥1.5) for data manipulation, matplotlib (≥3.6) and seaborn (≥0.11) for visualization, and NumPy (≥1.21) for numerical operations.

## 3 Key programmatic elements

**Figure 2:**
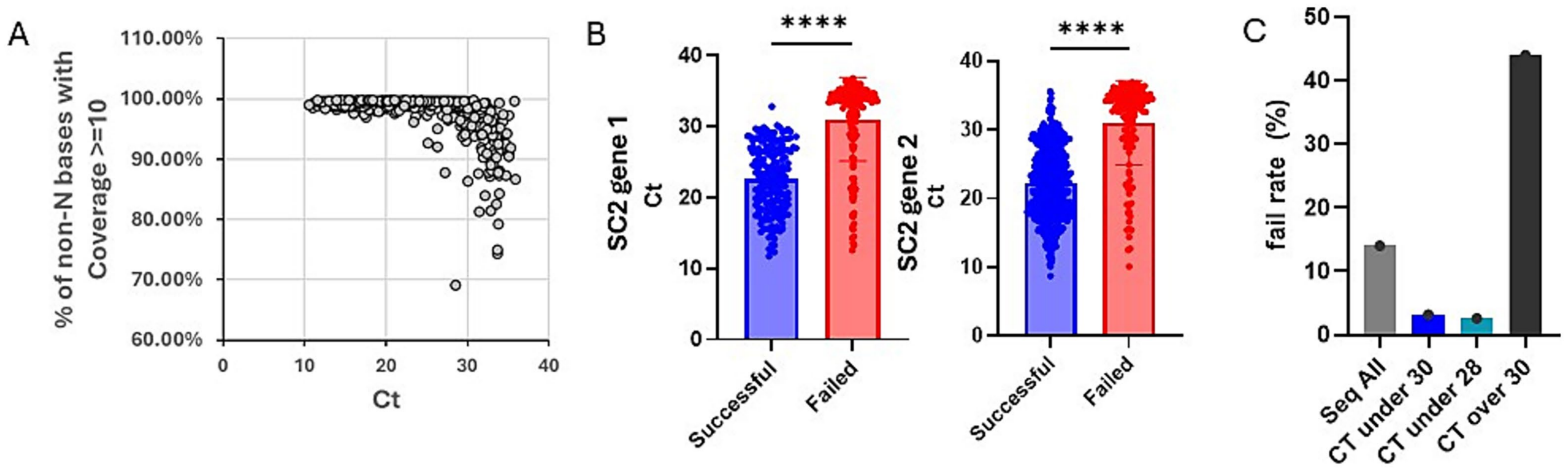
Simulated data suggests Ct restriction is cost effective but does not capture all variants in circulation. **(A)** Chart showing the Ct values of 811 samples vs. a measure of genome coverage, % of non-N bases with coverage >/= 10. **(B)** Chart showing the Ct values for the two PCR gene targets, Orf1 and N gene, of SARS-CoV-2 positive samples. Successful samples are shown in blue and the failed in red. Chart was generated on Graphpad Prism and statistical significance was determined by student *t*-test. **(C)** Chart showing sequencing fail rate for different sample sub-population based on Ct. Referenced data was based on 948 samples collected and sequenced between June through August 2022. Chart was generated on Graphpad Prism and statistical significance was determined by student t-test.

To proceed, we designed a two-phase study looking at our past sequencing data retrospectively and evaluating our SCQC+ approach prospectively (Figure 1C). In the retrospective phase, we analyzed sequencing data from the SC PHL collected from June through August 2022 during a period when all samples were sequenced regardless of Ct. Plotting the Ct of all sequenced samples during this time period against a sequence quality metric suggested that samples with Ct over 30 resulted in reduced coverage (Figure 2A). Samples that successfully completed the sequencing process had a mean Ct of 22.4 and 22.6 for two SARS-CoV-2 PCR gene targets, Orf1 and N gene, compared to 31.1 and 31.0 for samples that failed the sequencing (Figure 2B). Student t-test also confirmed that the differences between the Ct-values of successful and failed samples was statistically significant.

By excluding data from samples above a desired threshold, we could simulate the impact of using Ct-restriction to select samples for sequencing. Next, we show that sequencing only samples with Ct under 30 or Ct under 28 would have drastically reduced our sequencing fail rate from 13.8 to 3.2 and 2.6%, respectively (Figure 2C). Looking at the impact of these approaches on the number of unique variants detected, we determined that a Ct-under-30 approach would have identified 96% and a Ct-under-28 approach only detected 80% of variants in our datasets (Table 1).

**Table 1:** Overview of study findings.

Study phase	Sample selection approaches	Total samples sequenced (# samples eligible for seq)	Average Quality indicator for sequence run (% Runs passing filter/% coverage > = 10X)	# of distinct variant lineages detected	Variant capture	Fail rate	Cost per distinct variant lineage detected#	# of unique variant lineages in samples of Ct > 30	Cost to detect each novel variant not found in samples with CT < 30
Retrospective Phase	Seq All	948	58.2%/98.4%	30	100%	14%	$3,867	1	N/A
	Ct-under-30 simulated	691	N/A	29	96.7%	3.2%	$2,916	N/A	N/A
	Ct-under-28 simulated	624	N/A	24	80%	2.6%	$3,182	N/A	N/A
	Ct-over-30 simulated	248	N/A	19	63.7%	44%	$1,597	1	$30,350#
Prospective Phase	Ct-under-30	241	73.9%/99.1%	37	N/A	4.6%	$797	N/A	N/A
	SCQC+	572	88.5%/99.7%	79	N/A	2.1%	$903	2	N/A
	SCQC+ (Ct > 30) simulated	35 (84)**	N/A	18	N/A	22.9%	$278	2	$3,319*

**The number in parenthesis represents the number of samples with CT > 30 that were processed through the additional Agilent TapeStation QC, of which 35 passed the QC, and were sequenced.

*Calculated by adding the cost of processing 84 samples through the Tapestation QC at $15.67/sample and the cost of sequencing 35 samples that completed sequencing divided by the 2 novel variants detected.

# Cost was calculated by multiplication of total samples sequenced by the cost in reagents for library prep and sequencing of a sample ($122.38) divided by the number of distinct variant lineages found.

Next, we considered the cost effectiveness of both Ct-restriction approaches compared to sequencing all samples. We determined that either sequencing based on a Ct-under-30 selection criteria was more cost effective than a “Seq All” or Ct-under-28 approach. We also determined that amongst samples with high Ct (Ct > 30), though 19 distinct variants were detected, one variant found in a lone sample was detected which was not found among samples with Ct < 30. Detecting that rare variant would have required sequencing 241 high Ct samples at a 44% fail rate at the cost of over $30,000 (Table 1).

Altogether, the retrospective study suggested that Ct-over-30 approach to selecting samples for sequencing was more cost effective than sequencing all collected samples while enabling the capture of most but not all variants within our surveillance samples.

**Figure 3:**
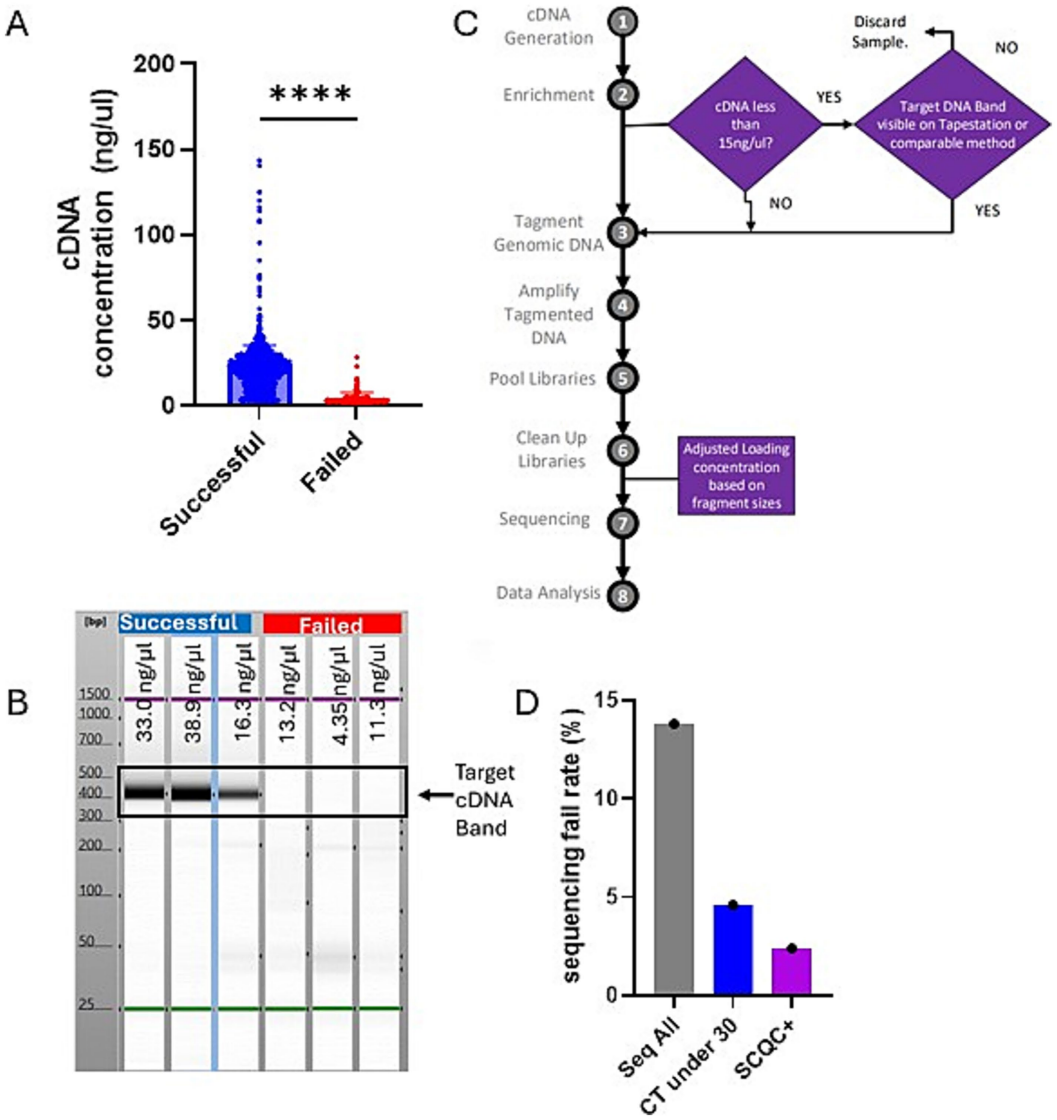
South Carolina Quality Control-plus method compared favorably with Ct restriction decreasing fail rate while maximizing capture of novel variants. **(A)** Chart showing the concentration of samples post cDNA generation step of library preparation grouped by whether those sequences were ultimately successful or failed. Chart was generated on Graphpad Prism and statistical significance was determined by student *t*-test. **(B)** A representative Agilent TapeStation result suggesting that successful samples should all have a clear band indicating the presence of the target cDNA. **(C)** A schematic showing the changes we implemented to the standard Illumina library prep method which we refer to as SCQC+. **(D)** Chart showing sequencing fail rate based on different sequencing approaches. The Seq All fail rate is reproduced from the earlier figure for comparison.

The prospective phase of our study was initiated by the observation that successful samples tended to have significantly higher cDNA concentrations early in the library prep process (Figure 3A). Additionally running the cDNA on a Agilent TapeStation instrument, an automated agarose electrophoresis machine, showed that failed samples tended to lack the expected target cDNA band (Figure 3B).

By plotting the cDNA concentration data, we observed that most of our failed samples had a cDNA concentration less than 15 ng/μL though they passed the manufacturer’s QC threshold of 3.3 ng/μL. This indicated that the typical QC with the Qubit instrument was insufficient at excluding samples that will ultimately fail during sequencing. Thus, we wanted to evaluate whether we could optimize our sequencing outcomes by reflexing samples with cDNA lower than 15 ng/uL to additional QC by the Agilent TapeStation. This would exclude samples without the target band from further processing, this could be a more cost-effective way to do sequencing. We also implemented the Agilent TapeStation to measure fragment sizes of the libraries before sequencing to optimize the loading of the machines. These two changes to the standard protocol are what we refer to as the South Carolina’s Quality Control plus method, hereafter SCQC+ (Figure 3C).

We were able to determine that sample selection using the SCQC+ approach led to higher quality sequencing runs as measured by % Runs Passing Filter (%RPF) metric compared to the Ct-under-30 approach (Table 1). SCQC+ approach also resulted in a low fail rate of 2.1% compared to the 4.6% fail rate when following a Ct-under-30 approach (Figure 3D). Exploring whether this method helped in the successful sequencing of samples with high Ct, we discovered that over 25 samples were successfully sequenced with a Ct over 30 (not shown) with a fail rate of 22.9% compared to 44% fail rate of the unmodified sequencing process (Table 1). Among these high Ct samples, two unique variants not found in specimens with Ct < 30 were detected using SCQC+ at a cost of only $3,319 (Table 1). In sum, this data suggested that the SCQC+ approach compared favorably with the Ct-based approach enabling more comprehensive variant surveillance while maintaining low fail rates.

**Figure 4:**
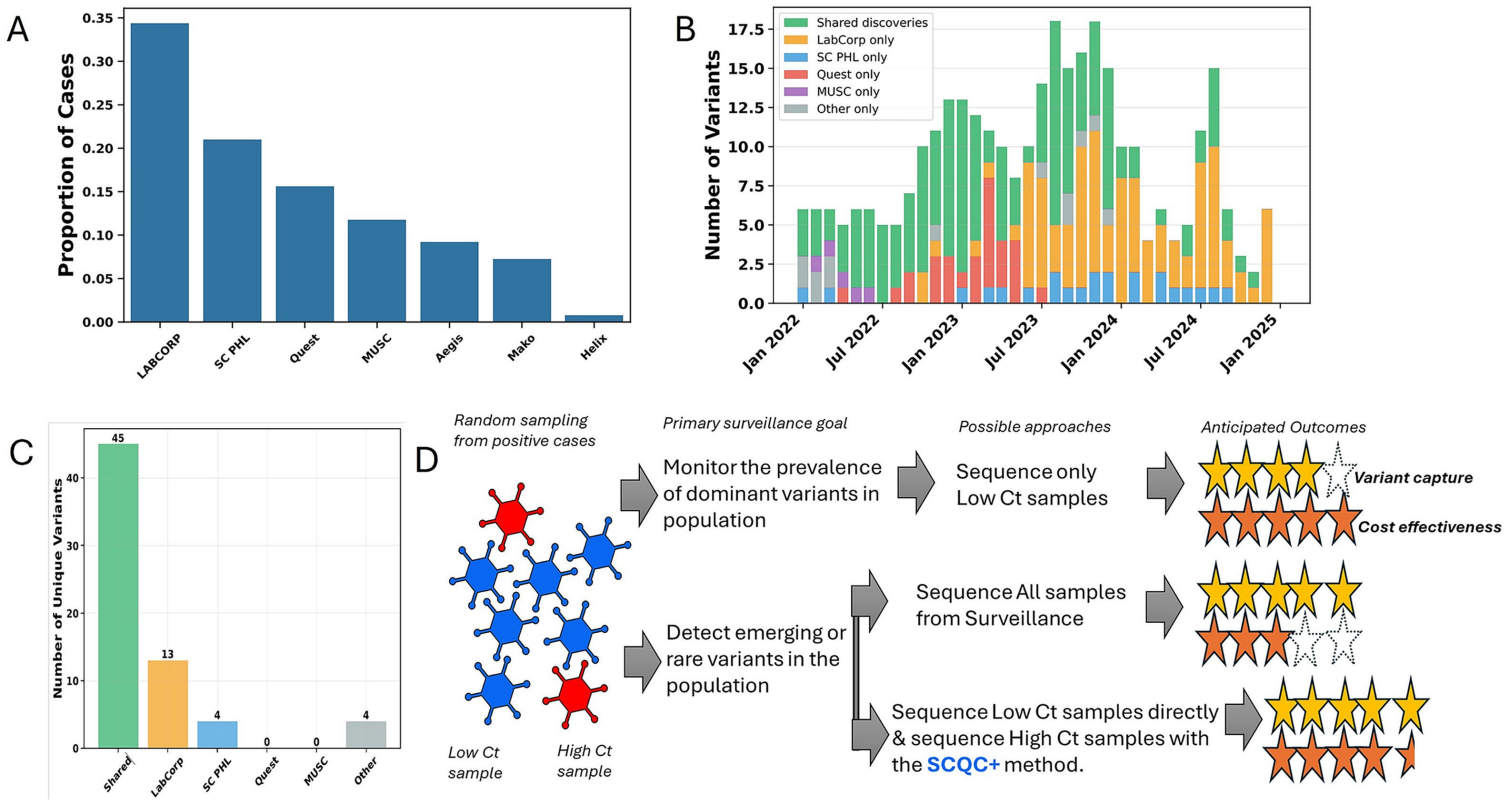
Commercial and clinical lab support throughout state contributed to a comprehensive variant surveillance network in South Carolina. **(A)** Chart showing the proportion of SARS-CoV-2 sequences that were provided by different sequencing laboratories during the study period. **(B)** Chart showing the number of variant lineages each month that were uniquely detected by different sequencing laboratories or found by more than one facility, labeled “Shared discoveries.” **(C)** Chart represents a summary of data from the entire study period for unique variants detected exclusively by different sequencing laboratory. **(D)** A summary of the findings of this case is shown graphically suggesting that the SCQC+ approach enables a more cost-effective approach for detecting rare variants in high Ct sample.

Next, we analyzed statewide variant surveillance data where many commercial and clinical laboratories contributed to the sequencing of SARS-CoV-2 positive cases throughout the state (Figure 4A). We were able to determine that the sampling of the various laboratories routinely identified unique variants not found by others in the same month (Figure 4B). While most variants were eventually found by different laboratories, Labcorp and SCPHL identified the most unique variants (Figure 4C). This suggested that even in a centralized public health agency, the support of commercial and clinical labs would be required for a comprehensive detection of rare emerging variants.

## 4 Discussion

According to the World Health Organization (WHO), there are two priority objectives for a variant surveillance program namely: monitoring the relative prevalence of variants across time and geographic areas and detecting variants circulating at low levels ((8)). Meeting such objectives enable decision makers to ensure that proposed interventions are adequate and effective to the variants circulating in their communities. Despite the failing prices of genomic sequencing technologies, the cost of WGS remains prohibitive and cost-effective methods for delivering on these goals of variant surveillance remain unclear.

Alternatives to WGS, such as multiplex PCR and wastewater surveillance (WWS), are available but can only deliver on one of the priority objectives for variant surveillance. While multiplex PCR is cheaper than WGS its utility is limited for monitoring the relative prevalence of variants of interest already defined by WGS. The accuracy of the test is also impacted by various factors including the spontaneous emergence of characteristic mutations from one lineage in an independent lineage; which may lead to an over-estimation of the prevalence of certain variants of concern (9) Lastly multiplex PCR cannot be used to detect new and emerging variants. Contrary to this relative paucity in variant detection with PCR, WWS enables the detection of a broad array of variants circulating in a population in a timely way (10). However, the significance of the WWS detected mutations remain unclear until confirmed in local clinical samples (11). As such while these technologies provide complementary data, WGS remains the gold standard to which they are compared and confirmed.

Ct value has been used as a proxy for the selection of high-quality samples for sequencing, culturing live viruses, and risk for viral transmission in households (3–6, 12). We reproduced the observation of Lu et al., that samples with Ct over 30 had reduced genome coverage. Our data also suggests the cost effectiveness of using a Ct-under-30 approach, but not Ct-under-28, for selecting samples when the objective is for monitoring the prevalence of non-rare variants, with successful samples on average having a Ct of 22 and failed samples with a Ct of 31.

However, other studies also demonstrated that 34% of secondary cases occurred in homes where the primary case had a Ct > 30 (5). Moreover, the Ct of a sample is impacted by a variety of factors like time from symptom onset, quality of collection method, or storage conditions and may not be an indicator of a reduced pathogenicity of the infecting virus stain (4, 13). Thus, in the search for the timely detection of VOCs/VOIs, all samples should be considered. Indeed, SCPHL data identified three instances of novel variants that appeared only in high Ct samples. However, the direct sequencing of high Ct samples lead to inordinate fail rates and reagent waste. Through the SCQC+ method, we were able to reduce the fail rate for sequencing high Ct samples in half while keeping the overall fail rate low.

The detection of low frequency variants requires large diagnostic testing volumes and sequencing volumes (8, 14). In South Carolina, we show that commercial and clinical labs were major contributors of sequence data asides from the public health agency. Population-wide sampling by different laboratories largely resulted in detection of shared variants but also produced unique variants, at least temporary on a month-to-month basis suggesting detection of emerging low-frequency variants. When the entire study period is considered, unique variants not identified by others were identified in Labcorp vs. SCPHL sequencing- suggesting the need for maintaining formalized partnerships between state, commercial, and clinical laboratories for a comprehensive surveillance of emerging variants before the next epidemic. In conclusion, our case study suggests cost effective approaches to molecular surveillance depending on the primary objectives of the surveillance program and introduces a Ct-independent method for optimizing the sequencing of high Ct samples (Figure 4D).

## 5 Study limitations and constraints

This case study has several limitations and constraints. For instance, in testing the SCQC+ method there were examples of samples that passed the Agilent TapeStation QC but eventually failed sequencing, suggesting the need for additional QC prior to sequencing. Additionally, the results presented came from the experience of one state public health agency and may need to be tailored to be applied to another. For example, at SCPHL we selected a threshold of 15 ng/ul for reflexing samples for additional QC. Therefore, it is possible that another laboratory seeking to implement the SCQC+ method may want to select a different threshold based on their own laboratory data. Finally, we used only one disease model and one sequencing method to test the cost effectiveness of SCQC+, it is possible that results may differ with other disease models or sequencing methods. However, since more than 50% of state public health agencies use the same ARTIC primers and 80% use Illumina sequencers, we believe that our study is highly relevant to many public health laboratories (15).

## Data Availability

The raw data supporting the conclusions of this article will be made available by the authors, without undue reservation.
